# Effect of Folic Acid on Hematological Changes in Methionine-Induced Hyperhomocysteinemia in Rats

**DOI:** 10.4103/0250-474X.56025

**Published:** 2009

**Authors:** M. N. Ansari, G. K. Nigam, Uma Bhandari

**Affiliations:** Department of Pharmacology, Faculty of Pharmacy, Jamia Hamdard (Hamdard University), New Delhi-110 062, India

**Keywords:** Folic acid, hematological changes, homocysteine, methionine, oxidative stress

## Abstract

The present study was designed to investigate the effect of folic acid on homocysteine, lipid profile and hematological changes in methionine-induced hyperhomocysteinemic rats. Hyperhomocysteinemia was induced by methionine (1 g/kg, p.o.) administration for 30 days. Biochemical and hematological observations were further substantiated with histopathological examination. The increase in homocysteine, total cholesterol, low density lipoprotein-cholesterol, very low density lipoprotein-cholesterol and triglycerides levels with reduction in the levels of high density lipoprotein in serum were the salient features observed in methionine treated toxicologic control rats (i.e. group II). Hematological observations of the peripheral blood smears of toxicologic rats also showed crenation of red blood cells membrane and significant (P<0.01) increase in total leukocyte count, differential leukocyte count and platelet counts with significant (P<0.01) decrease in the mean hemoglobin levels, as compared to vehicle control rats. Administration of folic acid (100 mg/kg, p.o.) for 30 days to methionine- induced hyperhomocysteinemic rats produced a significant (P< 0.01) decrease in the levels of homocysteine, total cholesterol, low density lipoprotein-cholesterol, very low density lipoprotein-cholesterol and triglycerides with significant (P< 0.01) increase in high density lipoprotein-cholesterol levels in serum when compared with toxicologic control rats. The present study, for the first time, investigates the effect of folic acid treatment on hematological changes in rats with methionine-induced hyperhomocysteinemia.

Cardiovascular disease is the most common cause of death in industrialized countries such as the US, and is on the rise in developing countries too. The National Heart, Lung, and Blood Institute of the National Institutes of Health has identified many risk factors for cardiovascular disease, including an elevated LDL-cholesterol level, high blood pressure, a low HDL-cholesterol level, obesity and diabetes[1]. In recent years, researchers have identified another risk factor for cardiovascular disease, an elevated homocysteine level. Homocysteine is an amino acid normally found in blood, but elevated levels have been linked with coronary heart disease and stroke[2–5]. Elevated homocysteine levels may impair endothelial vasomotor function, which determines how easily blood flows through blood vessels[6]. High levels of homocysteine also may damage coronary arteries and make it easier for blood clotting cells called platelets to clump together and form a clot, which may lead to a heart attack[2].

Although its physiopathological mechanisms are complex and not fully understood, much evidence suggests that hyperhomocysteinemia induces vascular damage because of the highly reactive thiol group in homocysteine that it is readily oxidized leading to the formation of homocysteine, homocysteine mixed disulfides and homocysteine thiolactone. During these oxidative processes, several reactive species are generated[7]. The oxidation of homocysteine promotes the oxidation of low density lipoprotein cholesterol[8], which causes injury to vascular endothelial cells[9,10] and leads to endothelial dysfunction[11].

Methionine is the only dietary source of homocysteine, which disrupts and interfere endothelial integrity[12]. Yen and Lau[13] have used methionine in a dose of 1 g/kg body weight to induce hyperhomocysteinemia in hypertensive rats. Excessive intake of methionine causes typical hematological changes: excess promotes methemaglobin accumulation and Heinz-body formation in erythrocytes and causes morphological changes in erythrocytic membrane which leads to hemolytic anemia and to morphological changes[14]. A deficiency of folate, vitamin B_12_ or vitamin B_6_ may increase blood levels of homocysteine, and folate supplementation has been shown to decrease homocysteine levels and to improve endothelial function[15–17]. At least, one study has linked low dietary folate intake with an increased risk of coronary events[18].

Evidence supports a role for supplemental folic acid for lowering homocysteine levels; however, this does not mean that folic acid supplements will decrease the risk of cardiovascular disease. Clinical intervention trials are underway to determine whether supplementation with folic acid, vitamin B_12_, and vitamin B_6_ can lower risk of coronary heart disease. It is premature to recommend folic acid supplementation for the prevention of heart disease until results of ongoing randomized, controlled clinical trials positively link increased folic acid intake with decreased homocysteine levels and decreased risk of cardiovascular disease. The present study was designed to investigate the effect of folic acid against L-methionine-induced hyperhomocysteinemia, hyperlipidemia and hematological changes in rats.

## MATERIALS AND METHODS

Methionine and folic acid were obtained from CDH, Mumbai. All other chemicals used were of analytical grade. Double distilled water was used for all biochemical assays. The experimental protocol was approved by the Institutional Animal Ethics Committee (IAEC) of Hamdard University, New Delhi, which is registered with Committee for the Purpose of Control and Supervision of Experiments on Animals (CPCSEA), Government of India, India (Registration no. 173/CPCSEA, dated 28 January, 2000). Wistar rats of either sex (150 to 200 g), were procured from the Central Animal House Facility, Hamdard University, New Delhi and acclimatized under standard laboratory conditions at 25±2°, 50±15% RH and normal photoperiod (12 h light:dark cycle) for 7 days. Commercial rat pellet diet (Nav Maharastra Chakan Oil Mills Ltd, Delhi, India) and water were provided *ad libitum*. Adequate measures were taken to minimize pain or discomfort, and that the experiments were conducted in accordance with international standards on animal welfare as well as being compliant with local and national regulations.

### Methionine-induced hyperhomocysteinemia:

The rats were divided into three groups of eight each. The first group served as vehicle control and 1% Tween 80 in normal saline (2 ml/kg, p.o.) was given orally for 30 days. The second group was toxicologic control and treated with L-methionine (1 g/kg, p.o.) for 30 days. The third group was folic acid treated group and folic acid (100 mg/kg, p.o.) was administered along with L-methionine (1 g/kg, p.o.) for 30 days.

### Biochemical analyses:

The blood was collected from the retro-orbital plexus of all the groups of overnight fasted rats using micro capillary tube on 31^st^ day. Serum was separated for biochemical estimations of homocysteine[19], HDL-C[20], total cholesterol[21] and triglycerides levels[22].

### Hematological studies:

The whole blood was used for estimation of haemoglobin levels and for the mounting of blood smears slides to investigate the hematological changes in rats viz. platelet count, total leucocyte count and lymphocyte count by using standard techniques[23]. The stained slides were studied under the low and high power objectives of the microscope for the study of morphology of white blood cells and differential leucocyte count (DLC). The slides were placed on a fixed stage and two drops of cedar wood oil were placed on the stained smear at a point about 2 cm from the start of the film.

### Statistical analysis:

All data were expressed as mean±SEM. The statistical analysis was performed using analysis of variance (ANOVA), followed by Dunnet ‘t’ test. Differences were considered statistically significant at P value<0.01.

## RESULTS

Table 1 shows the effect of folic acid on the homocysteine levels. Significant (P<0.01) increases in homocysteine levels were produced in rats treated with methionine (group II) when compared with vehicle control rats (group I). Oral administration of folic acid at dose of 100 mg/kg in hyperhomocysteinemic rats produced a significant (P<0.01) reduction in methionine-induced elevations of homocysteine levels (group III) as compared to toxicologic control rats (group II).

**TABLE 1:** EFFECTS OF FOLIC ACID ON SERUM HOMOCYSTEINE, TC, HDL-C AND TGs LEVELS IN METHIONINE-INDUCED HYPERHOMOCYSTEINEMIA

Treatment	Homocysteine (μg/ml)	TC (mg/dl)	HDL-C (mg/dl)	Triglycerides (mg/dl)
Vehicle control	8.75±0.25	117.37±3.64	43.15±2.73	71.42±3.21
Methionine (1 g/kg, p.o.)	22.55±1.85*	189.87±4.86*	17.77±1.54*	197.70±5.93*
Methionine (1 g/kg, p.o.) + folic acid (100 mg/kg, p.o.)	10.43±0.60#	141.31±4.33#	42.16±1.63#	102.63±2.14#

Values are mean±SEM, (n=8)

* P<0.01 when compared with vehicle control group

# P<0.01 when compared with methionine group; TC is total cholesterol, HDL-C is high density lipoprotein cholesterol and TGs stands for triglycerides

Tables 1 and 2 shows the effect of folic acid on serum lipid profile. Total cholesterol, triglycerides, LDL-C and VLDL-C levels were significantly (P<0.01) increased along with significant (P<0.01) decrease in HDL-C levels in rats treated with methionine (group II) when compared with vehicle control rats (group I). Folic acid treatment in methionine- induced hyperhomocysteinemic rats significantly (P<0.01) decreased the total cholesterol, triglycerides, LDL-C and VLDL-C levels and increased the HDL-C levels in serum as compared to toxicologic control rats (group II).

**TABLE 2:** EFFECT OF FOLIC ACID ON SERUM LDL-C, VLDL-C AND ATHEROGENIC INDEX IN METHIONINE- INDUCED HYPERHOMOCYSTEINEMIA

Treatment	LDL-C (mg/dl)	VLDL-C (mg/dl)	Atherogenic Index	
			
			(TC/ HDL-C)	(LDL-C/HDL-C)
Vehicle control	59.94±1.00	14.28±0.64	2.78±0.16	1.49±0.13
Methionine (1 g/kg, p.o.)	132.55±5.71*	39.53±1.18*	10.66±0.77*	8.42±0.97*
Methionine (1 g/kg, p.o.) + folic acid (100 mg/kg, p.o.)	78.58±4.69#	20.52±0.43#	3.69±0.13#	2.47±0.28#

Values are mean±SEM, (n=8)

* P<0.01 when compared with vehicle control group

# P<0.01 when compared with methionine group; LDL-C is low density lipoprotein cholesterol and VLDL-C denotes very low density lipoprotein cholesterol

The mean platelets counts, total leukocyte counts (TLC), polymorphonuclear cells (PMN), and lymphocyte counts were significantly increased with significant decrease in blood hemoglobin levels in methionine-treated group (i.e. toxicologic control rats, group II) as compared to the vehicle control (i.e. group I) rats. While methionine treatment did not cause any significant change in monocyte count as compared to the vehicle control rats. Further, treatment with folic acid to methionine-treated rats (i.e. group III) significantly decreased the elevated platelets, TLC, PMN and lymphocyte counts as compared to the toxicologic control group (i.e. group II) rats and no significant changes were observed in monocyte count and blood hemoglobin levels (Tables 3 and 4).

**TABLE 3:** EFFECT OF FOLIC ACID ON HEMOGLOBIN LEVELS, PLATELETS AND TLC COUNT IN METHIONINE-INDUCED HYPERHOMOCYSTEINEMIA

Treatment	Hemoglobin (g/dl)	Platelets counts (Lacs/cu mm)	TLC thousands/cu mm)
Vehicle Control	14.35±0.44	0.85±0.02	6800±0.87
Methionine (1 g/kg, p.o.)	12.37±0.25*	1.10±0.02*	8900±0.77*
Methionine (1 g/kg, p.o.) + folic acid (100 mg/kg, p.o.)	14.45±0.34^ns^	0.90±0.25#	7300±0.12#

Values are mean±SEM, (n=8)

* P<0.01 when compared with vehicle control group

# P<0.01 when compared with methionine group

ns non significant, when compared with methionine group; TLC- Total Leucocytes Count

**TABLE 4:** EFFECT OF FOLIC ACID ON PMNS CELLS, LYMPHOCYTES AND MONOCYTES IN METHIONINE- INDUCED HYPERHOMOCYSTEINEMIA

Treatment	PMNs cells (%)	Lymphocytes (%)	Monocytes (%)
Vehicle control	09±1.000	82±0.500	05±1.002
Methionine (1 g/kg, p.o.)	13±1.3148*	95±1.250*	06±1.308ns*
Methionine (1 g/kg, p.o.) + folic acid (100 mg/kg, p.o.)	10±0.275#	84±1.555#	06±0.819ns#

Values are mean±SEM, (n=8)

* P<0.01

ns* (non significant) when compared with vehicle control group

# P<0.01

ns# (non significant) when compared with methionine group; PMNs- Polymorphonuclear cells.

**Fig. 1:**
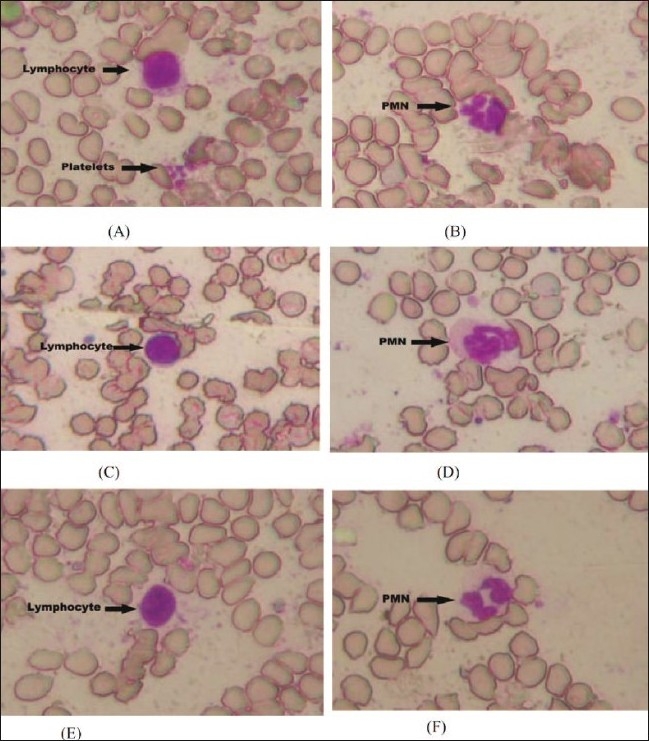
Photomicrographs of blood smear from the vehicle control, pathogenic control and folic acid-treated rats. A and B represents vehicle control group showing lymphocyte with uniformly stained oval nucleus and a thin surrounding rim of cytoplasm and a small group of blood platelets is seen in the lower portion of image. Normal polymorphonuclear cells (PMNs) and erythrocytes with clearly brought out normal cell membrane and cytoplasm. C and D represents methionine treated rats showing red blood cells crenation and other morphological changes in erythrocytic membrane. Lymphocyte with uniformly stained nucleus showing marked inflammatory infiltrate with oedema. A small group of aggregated platelets is seen in the lower portion of the image. Polymorphonuclear leukocyte with multi-lobed nucleus. E and F represents methionine+folic acid (100 mg/kg) treated rats showing lymphocyte with uniformaly stained oval nucleus and a thin surrounding rim of cytoplasm with no crenation of erythrocytic membrane suggesting protection of RBCs by folic acid. Polymorphonuclear leukocyte with multi-lobed nucleus. Original magnification X 400.

Photomicrograph of vehicle control rats (i.e. group I) revealed a normal architecture with regular morphology of various blood cells viz. erythrocytes, lymphocytes, polymorphonuclear cells and platelets distributed and stained uniformly (figs. 1A and 1B). Toxicologic control i.e. group II rats showed crenation (shrinkage of erythrocytes giving notched appearance) and other morphological changes in erythrocytic membrane. Also, there were groups of aggregated platelets in the lower portion of the image. Polymorphonuclear cells with multilobed nucleus and lymphocytes with uniformly stained nucleus were also present (figs. 1C and 1D). Folic acid treated group showed lymphocyte with uniformly stained oval nucleus and a thin surrounding rim of cytoplasm with no crenation of erythrocytic membrane suggesting protection of RBCs by folic acid. Polymorphonuclear leukocyte with multi-lobed nucleus (figs. 1E and 1F) and lymphocytes with uniformly stained nucleus were also present.

## DISCUSSION

Hyperhomocysteinemia has recently been emerged as an independent risk factor for development of coronary, cerebrovascular and peripheral arterial occlusive disease[24]. Recent epidemiological data have shown that hyperhomocysteinemia can be detected in 20 and 40% of patients with coronary artery disease and cerebrovascular disease, respectively[25], which is in agreement with homocysteine theory of arteriosclerosis proposed by McCully and Wilson in 1969, who reported that hardening of the arteries was directly related to the amino acid, homocysteine[26].

Numerous studies have demonstrated that hyperhomocysteinemia produces endothelial damage and dysfunction increasing the risk of atherogenesis and thrombogenesis through oxidative mechanisms[27–29]. Homocysteine, a thiol containing amino acid derived from demethylation of dietary methionine, may generate partially reduced ROS that are able to stimulate the lipid peroxidation involved in atherosclerotic process. Thus, an imbalance in dietary methionine may contribute to the development of atherosclerosis by increasing homocysteine levels[30].

Methionine administration for a period of 30 days induced a significant hyperhomocysteinemia and haematological changes in rats (P<0.01) increase in homocysteine, TC, TGs, LDL-C and VLDL-C levels and decrease in HDL-C levels in serum in toxicologic control group rats (i.e. group II) as compared to vehicle control group (i.e. group I). The increase in the homocysteine levels with methionine administration is in agreement with the findings of Kapoor *et al*.[31] and Zulli *et al*[32].

Oral treatment with folic acid (100 mg/kg, p.o., Group III) for 30 days produced a significant antihyperhomocysteinemic and lipid lowering activities in methionine-induced hyperhomocysteinemia in rats. Therefore, our present study is in agreement with the findings of HLTC[33], Rydlewicz *et al*[34] and Lamers *et al*[35]. Furthermore, there was decrease in elevated platelet counts, TLC, PMN cells (i.e. neutrophils, eosinophils and basophils) and lymphocyte counts as compared to toxicologic control group (i.e. group II). However, monocyte count and blood hemoglobin levels were not significantly decreased as compared to toxicologic control group. Nicholas *et al*.[36] observed that leukocyte mediated changes in endothelial integrity and function may lead to the vascular disease seen in individuals with elevated plasma homocysteine.

The involvement of peripheral blood monocytes in the development of atherosclerosis is now well established[37]. Animal models of hypercholesterolaemia-related atherosclerosis suggest that monocyte characteristics and behavior may be altered. Leucocyte total white cell, monocyte absolute and differential percentage counts are increased in cholesterol fed rats[38,39]. Our study shows that methionine fed to normal rats (not cholesterol fed to normal rats) resulted in increase in monocyte count but we could not find a statistically significant increase, while folic acid treatment decreased the monocyte but again the decrease was not statistically significant. This finding is in contrast to the study by Bath *et al.*[40] who studied the alterations in monocyte characteristics in familial hypercholesterolaemic patients, not hyperhomocysteinemic patients, and reported the increased involvement by monocytes in hyperhomocysteinemia-induced atherogenesis.

The results of biochemical observations were supplemented by hematological examination of rat's blood smear of all the groups. No alterations of morphological appearance of leucocytes could be observed in any of the groups examined. Crenation of RBCs (shrinkage of erythrocytes giving notched appearance) cell membrane in methionine-treated rats was indicative of hemolytic anemia (figs. 1C and 1D). Klavins *et al*[14] and Benevenga *et al*.[41] have reported that excessive intake of methionine causes typical hematological changes: excess promotes Heinz-body formation in erythrocytes and causes morphological changes in erythrocytic membrane which leads to hemolytic anemia. However, folic acid treatment (i.e. group III) could not fully protect the red blood cell membrane and crenation of few red blood cells (figs. 1E and F).

We demonstrated in our study that oral treatment with folic acid exhibit antihyperhomocysteinemic and lipid lowering activity with reversal of many hematological changes induced by methionine. Folic acid may, thus, be a new treatment option for patients with hyperhomocysteinemia, as homocysteine emerges as a risk factor for cardiovascular diseases.
